# Tuberculous Meningitis in Pediatric Patients: Diagnosis, Clinical Course, and Outcomes in the Modern Era

**DOI:** 10.3390/pathogens15090928

**Published:** 2026-09-03

**Authors:** Irina Vasilyeva, Nadezhda Klevno, Aleksey Kazakov, Aziza Pakhlavonova, Alena Prikhodko, Ekaterina Sokolskaya, Elizaveta Zakharova, Ksenia Rudakova, Alexandr Chuishchev, Inga Enilenis, Ivan Martel

**Affiliations:** 1 National Medical Research Center of Phthisiopulmonology and Infectious Diseases, Ministry of Health of the Russian Federation, Moscow 127473, Russiak.rudackowa2017@yandex.ru (K.R.); 2 Pirogov Russian National Research Medical University, Ministry of Health of the Russian Federation, Moscow 117997, Russia; 3 Moscow Regional Research and Clinical Institute Named After M.F. Vladimirsky, Moscow 123592, Russia; 4 Sechenov First Moscow State Medical University (Sechenov University), Moscow 119991, Russia

**Keywords:** tuberculous meningitis, encephalitis, children, multidrug resistance (MDR-TB), complications, BCG vaccination, outcomes

## Abstract

Tuberculous meningitis (TBM) is a critically severe form of extrapulmonary tuberculosis in pediatric patients, presenting a significant diagnostic challenge during the early stages of clinical manifestation. Delayed recognition of meningeal tuberculous inflammation leads to the involvement of the brain parenchyma (encephalitis) and may result in irreversible neurological sequelae. Objective: To evaluate the clinical features, the spectrum of complications, and the acute outcomes of tuberculous meningitis in pediatric patients managed at a tertiary reference center. Materials and Methods: A retrospective observational study of 21 medical records was conducted for pediatric patients aged 3 months to 17 years (mean age: 6.7 ± 5.8 years) with a confirmed TBM diagnosis. The study included pediatric patients treated in the inpatient pediatric department (*n* = 6) or evaluated through the national telemedicine consultation system (*n* = 15) between 2017 and 2025. Results: A history of contact with a tuberculosis patient was established in 62% (13/21) of cases. At initial presentation, fever was recorded in 67% (14/21) of patients, nausea and/or vomiting in 52% (11/21), headache in 33% (7/21), and meningeal signs in 38% (8/21). The majority of patients (52%, 11/21) had not received BCG vaccination. *Mycobacterium tuberculosis* (MTB) was microbiologically confirmed in 19 patients (90.5%). MTB DNA was detected in the cerebrospinal fluid (CSF) in 71.4% of all patients (15/21; 78.9% of confirmed cases). Drug resistance (DR) was confirmed in 47.4% of confirmed patients (9/19; 42.9% of the total cohort), comprising MDR (15.8%, 3/19), pre-XDR (15.8%, 3/19), and XDR (15.8%, 3/19) strains. Neurological complications included hydrocephalus in 47.6% (10/21), motor deficits in 38.1% (8/21), and epileptic syndrome in 19.0% (4/21). In-hospital mortality was 0%, while four patients (19.0%) with critically severe irreversible brain damage were transitioned to palliative care. Conclusion: In this tertiary pediatric cohort, TBM presented predominantly as a severe form of meningoencephalitis with extensive brain parenchymal involvement and high rates of systemic dissemination, frequently complicated by hydrocephalus, motor deficits, and seizures. Epileptic seizures showed an exploratory statistical trend toward an association with unfavorable neurological outcomes (*p* = 0.052). These findings underscore the urgent need to optimize early diagnostic strategies within general healthcare networks.

## 1. Introduction

Tuberculous meningitis (TBM) is the most severe form of extrapulmonary tuberculosis (TB) in children, resulting from the hematogenous dissemination of *Mycobacterium tuberculosis* (MTB), to the leptomeninges, where it triggers a specific inflammatory response [1]. TBM remains the most debilitating and life-threatening form of extrapulmonary TB in pediatric practice. When diagnosis is delayed, the case fatality rate can exceed 60%, and the incidence of neurological complications can reach 50% or higher [2]. Despite an overall decline in TB incidence across the Russian Federation, the incidence rate in 2023 among children aged 0–14 years was 6.7 per 100,000, and among adolescents aged 15–17 years, it stood at 12.7 per 100,000 age-matched population. Concurrently, TBM is diagnosed in 0.5–1% of cases (mean: 0.86%) of all newly diagnosed pediatric TB patients [3,4].

Early detection of MTB is challenging and heavily relies on the clinical index of clinical suspicion among general practitioners, as pediatricians are typically the first point of contact for these patients.

In contemporary clinical practice, the classic triad of meningeal symptoms (headache, vomiting, and fever) is increasingly absent or attenuated. Instead, atypical, indolent courses are frequently observed, where diffuse cerebral symptoms predominate over classic meningeal manifestations [2,5]. Furthermore, in infants and young children, the clinical presentation is often dominated by equivalents of meningeal signs such as hyperkinesis, paroxysmal events, and inconsolable crying, necessitating optimized diagnostic algorithms [2,5]. In addition, an increasing proportion of tuberculosis cases is caused by multidrug-resistant (MDR) strains of MTB [2,6,7]. Research indicates that TBM caused by MDR strains is associated with a more severe clinical course, delayed resolution of neurological symptoms, and the necessity to prescribe anti-tuberculosis drugs with limited blood–brain barrier (BBB) penetration, all of which may adversely affect the prognosis [8]. In addition, an increasing number of children remain unvaccinated with BCG or BCG-M, resulting in a growing subpopulation of immunologically unprotected young children. In these individuals, TB often presents as a disseminated disease with a high risk of rapid progression to meningoencephalitis [9,10]. Concurrently, the diagnosis of TBM remains highly challenging due to the paucity of cerebrospinal fluid (CSF) findings during the early stages of the disease. Specifically, lymphocytic pleocytosis often does not become apparent until days 5–7 of illness, and the sensitivity of smear microscopy remains low [9,10,11]. Although the implementation of molecular genetic methods (PCR) reduces the detection time for MTB DNA, their sensitivity remains far from 100% and varies according to the stage of the disease [12,13]. For instance, a study by Sehgal et al. (2023) demonstrated that the sensitivity of PCR methods (Pab PCR and IS6110 PCR) in TBM was 82% and 76%, respectively, failing to reach absolute values even under optimal conditions [12]. According to Ghimire et al. (2023), the sensitivity of GeneXpert MTB/RIF in CSF analysis ranges between 50% and 80%, demonstrating that a diagnosis of TBM cannot be excluded on the basis of a negative PCR result alone [13].

Taken together, these factors underscore the urgent relevance of a comprehensive evaluation of the clinical and laboratory features, as well as the outcomes, of tuberculous meningitis in pediatric patients within modern tuberculosis care.

## 2. Materials and Methods

The study was conducted in accordance with the principles of biomedical ethics set forth in the 1964 Declaration of Helsinki and its subsequent amendments. The study protocol was approved by the Ethics Committee of Sechenov University (Moscow), Protocol No. 11–26 dated 21 May 2026.

This is a retrospective observational study (case series). The medical records of 21 pediatric patients with tuberculous meningitis (meningoencephalitis) were retrospectively analyzed. The cohort comprised all consecutive pediatric patients managed at the National Medical Research Center for Phthisiopulmonology and Infectious Diseases between 2017 and 2025: 6 patients (28.6%) treated directly in the Pediatric Tuberculosis Inpatient Department and 15 patients (71.4%) evaluated through the federal telemedicine consultation system from regional healthcare facilities (treated at their place of residence under center supervision). Female patients accounted for 57.1% (12/21) of the cohort. Children younger than 7 years comprised 66.7% (14/21), with those younger than 3 years accounting for 52.4% (11/21) (Table 1). The mean age was 6.7 ± 5.8 years (range: 3 months–17 years).

TBM diagnosis was verified according to standard uniform international consensus criteria [14]:

- Definite TBM (*n* = 15, 71.4%): Confirmed by identification of MTB directly in the cerebrospinal fluid (CSF) via real-time PCR (MTB DNA detected in CSF in 15/21, 71.4%) and/or liquid culture (BACTEC MGIT 960).
- Probable TBM (*n* = 6, 28.6%): Established based on the consensus scoring framework (score ≥ 12 with neuroimaging), which included 4 patients with microbiological evidence of active extra-CNS tuberculosis (pulmonary, gastric, or urinary) supporting the clinical diagnosis.

In this study, *meningoencephalitis* was defined as TBM accompanied by clinical or radiologic evidence of cerebral parenchymal involvement (focal motor deficits, refractory seizures, quantitative impairment of consciousness, brainstem/subcortical dysfunction, or parenchymal ischemia/tuberculomas on neuroimaging).

All patients underwent standardized diagnostic evaluation, including:

- Complete blood counts, routine urinalysis, and serum biochemical panels;
- Chest computed tomography (CT);
- Lumbar puncture with quantitative CSF analysis (total cell count, differential, total protein, glucose, CSF-to-blood glucose ratio, and chloride);
- Molecular genetic identification of MTB DNA in CSF and other biological specimens using validated commercial real-time PCR assays (‘AmpliSens^®^ MTB-complex-FL’, Central Research Institute of Epidemiology, Moscow, Russia, targeting the IS6110 insertion sequence and *regX3 gene;* and GeneXpert^®^ MTB/RIF, Cepheid, Sunnyvale, CA, USA, targeting the rpoB core region);
- Mycobacterial culture and phenotypic drug susceptibility testing (DST) on liquid media using the automated BACTEC™ MGIT™ 960 system (Becton Dickinson, Sparks, MD, USA) for first-line and second-line drugs, supplemented by molecular line-probe assays;
- Neuroimaging (contrast-enhanced brain MRI and/or CT);
- Pre-referral immunodiagnostic data extraction (Mantoux test with 2 TU PPD-L and recombinant tuberculosis allergen [RTA; Diaskintest^®^, Generium, Russia]) from available primary records;
- Multidisciplinary specialist assessments (ophthalmologist, neurologist, neurosurgeon).

Adverse clinical outcomes evaluated at the completion of acute in-hospital therapy included critically severe irreversible brain damage requiring transition to palliative care (persistent vegetative state, severe tetraparesis, optic atrophy, and coma), persistent neurological impairment (spastic paresis/paralysis, psychomotor retardation, secondary seizure disorder, optic atrophy, shunt-dependent hydrocephalus), and clinical improvement.

Statistical processing was performed using Statistica 12.0 (StatSoft Inc., Tulsa, OK, USA). Quantitative variables were summarized as mean ± standard deviation (M ± SD) or median with interquartile range (IQR: 25th–75th percentiles) and ranges. Categorical variables were expressed as absolute numbers and percentages (%). Associations between categorical variables were analyzed using Fisher’s exact test. Odds ratios (OR) with 95% confidence intervals (95% CI) were calculated. A *p*-value < 0.05 was considered statistically significant.

A history of contact with a tuberculosis patient was established in 13 children (62%); in 7 of these cases (54%), MDR or pre-XDR strains were reported in the suspected source cases. Ten children (48%) had a history of BCG vaccination.

## 3. Results

Almost all children (20/21; 95.2%) were referred to tuberculosis departments from general healthcare network (GHN) facilities, indicating a high rate of initial medical consultations at non-specialized medical organizations. Only one patient (4.8%) was identified during contact tracing at a community tuberculosis dispensary.

BCG vaccination prevents the dissemination of tuberculosis and the development of meningitis in infants and young children aged under 3 years. As shown in Table 1, 81.8% (9/11) of patients in this age group who developed meningitis had not received BCG vaccination; furthermore, 10 of 11 children (91%) had a confirmed history of contact with a tuberculosis patient.

A significant number of patients had an irregular history of immunodiagnostic testing prior to the final diagnosis. Specifically, the Mantoux test with 2 TU was performed in only 9/21 patients (43%) during routine screening. Among these, positive results (an induration equal to or greater than 5 mm in at least one measurement) were observed in 7/9 patients (77.7%), while negative results (all measurements less than 5 mm or completely negative) were recorded in 2/9 patients (22.3%). The recombinant tuberculosis allergen (RTA) skin test was performed in only 10/21 patients (47%), even when tuberculosis was suspected. Positive results (the presence of an induration of any size or erythema greater than 5 mm) were found in 6/10 patients (60%), and negative results were noted in 4 patients (40%).

The clinical presentation was characterized by constitutional symptoms, such as fever (14 patients, 67%) and marked lethargy/weakness (9 patients, 43%). Nausea and/or vomiting were observed in 11 patients (52.4%), while cephalgia was reported in 7 patients (33.3%). Meningeal signs were documented at initial presentation in only 8 patients (38.1%), indicating an attenuated onset or the predominance of diffuse cerebral symptoms during the early stage. Motor impairments (limb weakness, gait instability, paresis) were documented in 8 patients (38.1%), and seizures were present in 4 patients (19.0%) (Table 2). The disease onset was most frequently subacute or acute, mimicking acute respiratory viral infections (ARVI) or gastroenteritis. At the peak of the disease, neurological manifestations included meningeal syndrome (nuchal rigidity, Kernig and Brudzinski signs, which were not always fully expressed, particularly in infants and young children), focal deficits, brainstem and subcortical syndromes, altered consciousness (ranging from stupor to grade I–II coma), and hydrocephalic syndrome. Quantitative cytological and biochemical CSF evaluation at initial lumbar puncture revealed characteristic abnormalities across all 21 patients (100%): CSF total cell count showed marked pleocytosis with a median of 185 cells/μL (IQR: 84–340; range: 24–680 cells/μL), with a pronounced lymphocytic predominance (median lymphocytic fraction: 78%, IQR: 65–88%). CSF total protein was significantly elevated, with a median of 1.95 g/L (IQR: 1.20–3.10 g/L; range: 0.82–5.40 g/L). CSF absolute glucose was decreased, with a median of 1.35 mmol/L (IQR: 0.90–1.80 mmol/L), resulting in a median CSF-to-blood glucose ratio of 0.28 (IQR: 0.18–0.36; range: 0.12–0.44). CSF chloride levels were reduced, with a median of 110.5 mmol/L (IQR: 104.0–116.0 mmol/L).

**Figure 1:**
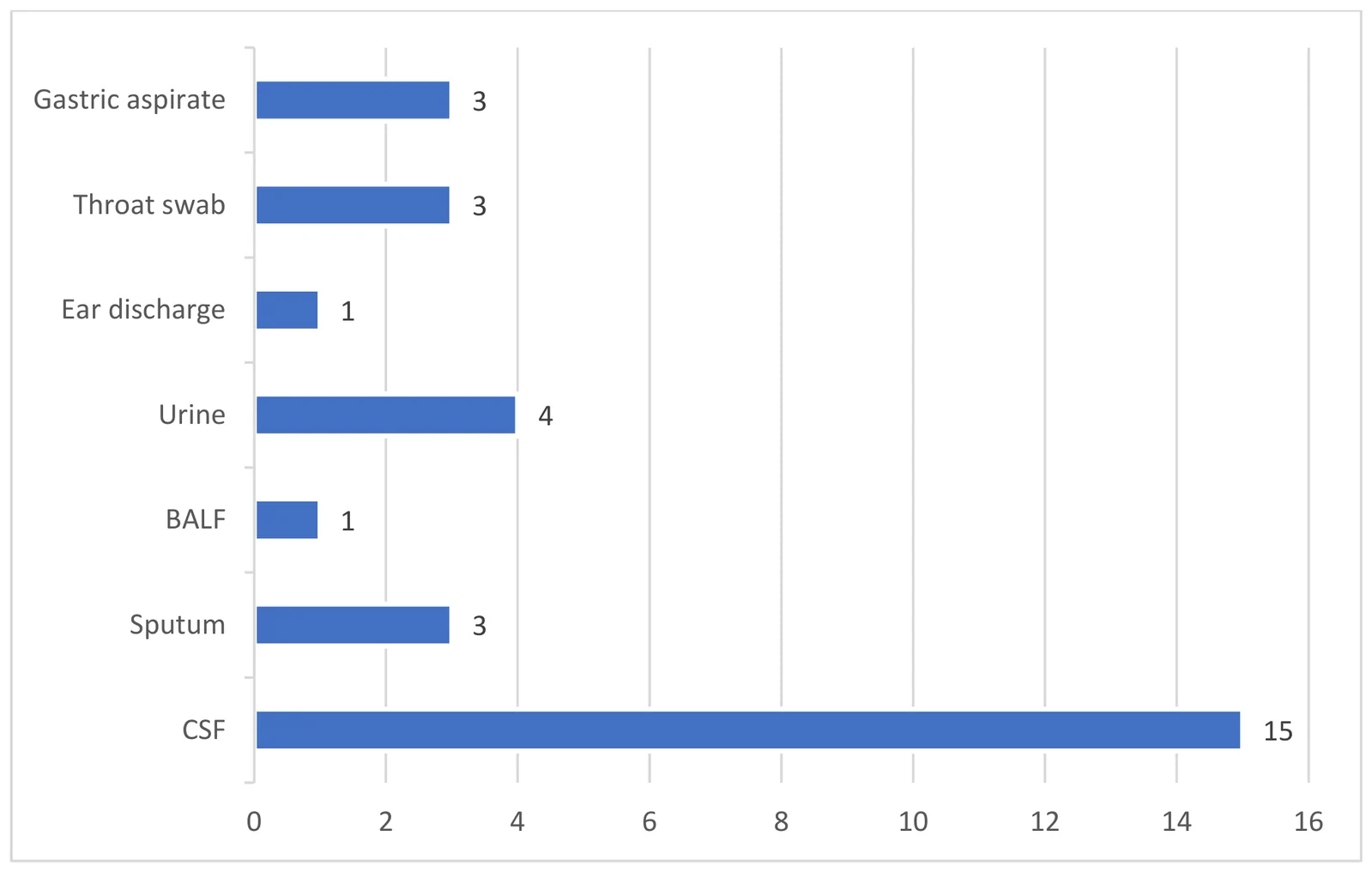
Detection rates of *Mycobacterium tuberculosis* across biological specimens in the study cohort (*n* = 21) BALF—bronchoalveolar lavage, CSF—Cerebrospinal Fluid.

The causative agent was definitively identified in 19 of 21 patients (90.5%), whereas microbiological findings were inconclusive in the remaining 2 patients. Specifically, MTB DNA was detected in the CSF of 15/21 patients (71.4% of the total cohort; 78.9% of confirmed cases) by real-time PCR, in urine samples of 4/21 (19.0%), and in sputum, gastric aspirates, or tracheal aspirates of 3/21 (14.3% each). MTB was also isolated from BALF in 1/21 (4.8%) and from ear discharge in 1/21 (4.8%) (Figure 1).

Drug susceptibility testing was available for 16 of the 19 microbiologically confirmed patients (culture DST remained pending/unavailable in 3 cases). Drug resistance (DR) was confirmed in 9 of 19 confirmed patients (47.4%; 42.9% of the total cohort), comprising multidrug-resistant (MDR; *n* = 3, 15.8%), pre-extensively drug-resistant (pre-XDR; *n* = 3, 15.8%), and extensively drug-resistant (XDR; *n* = 3, 15.8%) strains, while 7 patients (36.8%) harbored drug-susceptible strains (Table 3).

Neuroimaging (brain CT/MRI) revealed signs of hydrocephalus in 10/21 patients (47.6%), categorized as obstructive (3 patients, 14.3%), communicating (ex vacuo/compensatory) (2 patients, 9.5%), triventricular (1 patient, 4.8%), quadriventricular (1 patient, 4.8%), shunted hydrocephalus with slit ventricles (1 patient, 4.8%), trapped ventricle syndrome (1 patient, 4.8%), and mixed communicating hydrocephalus (1 patient, 4.8%). Structural brain changes were identified in 4/21 patients (19.0%), presenting as cystic-gliotic transformations, parabrainstem cysts, cortical subatrophy, and central pontine myelinolysis.

The primary neurological complications documented during the acute hospital stage, listed in descending order of frequency, were:

- Hydrocephalus: 10 of 21 patients (47.6%);
- Cranial nerve palsies and sensory deficits: 9 of 21 patients (42.9%);
- Motor deficits (paresis/paralysis): 8 of 21 patients (38.1%), presenting as spastic asymmetric tetraparesis, hemiparesis, inferior paraparesis, and diplegia;
- Respiratory failure: 5 of 21 patients (23.8%);
- Epileptic syndrome: 4 of 21 patients (19.0%), including focal, generalized tonic–clonic seizures, and epileptic spasms;
- Brainstem and subcortical syndromes: 4 of 21 patients (19.0%), including bulbar palsy and dystonic storms;
- Optic nerve atrophy: 3 of 21 patients (14.3%);
- Contractures and orthopedic deformities: 2 of 21 patients (9.5%).

Surgical Interventions: Neurosurgical management of hydrocephalus was required in 6 patients (28.6%), comprising external ventricular drainage, ventriculoperitoneal shunting, and Ommaya reservoir placement. Shunt dysfunction requiring revision occurred in 2 patients (9.5%).

Table 4 presents data on the time to diagnosis, BCG vaccination status, and treatment outcomes in 21 pediatric patients stratified according to the diagnostic period duration. The final diagnosis was established more than 14 days after initial symptoms onset in the majority of patients (57%). Diagnosis was achieved within 7 days in 24% of cases, and between 7 and 14 days in 19%. Treatment outcomes analysis demonstrated that, in the earliest diagnosis group (within 7 days), 80% of patients completed treatment with a favorable clinical response, defined as clinical and radiological improvement followed by transfer to dispensary monitoring. In contrast, among patients with a diagnostic delay exceeding 14 days, only 42% achieved a similarly favorable outcome. Furthermore, 33% of these children (4 patients) were transitioned to palliative care. These findings underscore the critical importance of early TBM detection, given the severe disease course and the limited efficacy of etiotropic therapy in the advanced stages.

According to the analysis of treatment outcomes, critically severe, irreversible brain injury was documented in 4 of 21 patients (19%), who were subsequently transitioned to palliative care. These patients presented with persistent vegetative state, profound tetraparesis, optic nerve atrophy, and coma.

In 6 of 21 children (29%), persistent neurological deficits remained, including spastic paresis/paralysis (hemiparesis or tetraparesis), psychomotor retardation, seizure disorder, optic nerve atrophy, and shunt-dependent hydrocephalus.

Clinical improvement was recorded in 10 of 21 (48%) patients. However, residual sequelae (such as hemiparesis and hydrocephalus) persisted even within this subgroup.

Correlation analysis between complications and treatment outcomes (palliative care vs. clinical improvement) demonstrated a weak trend toward a higher incidence of hydrocephalus in patients with a palliative outcome. However, no statistically significant association was found (Fisher’s exact test: *p* = 0.105 for hydrocephalus, *p* = 0.266 for paresis).

Concurrently, for epilepsy and seizures, the *p*-value was 0.052, which closely approaches the significance threshold of 0.05. This suggests a potential trend wherein seizures occur more frequently in patients with a palliative outcome (75% vs. 11%). The odds ratio was OR = 24 (95% confidence interval [CI]: 1.02–1364); however, the confidence interval is extremely wide due to the small sample size, highlighting the need for further validation in a larger patient cohort. Furthermore, an aggregate analysis of the relationship between any of the three major complications (hydrocephalus, paresis, or seizures) and clinical outcomes revealed no statistically significant association (*p* = 0.228), which is likely attributable to the limited sample size and missing data regarding certain patients’ vaccination status and long-term outcomes.

In summary, no statistically significant associations were identified between BCG vaccination status and clinical outcomes, nor between vaccination and most complications. However, a distinct trend was noted toward a higher seizure frequency in unvaccinated patients (*p* = 0.092) and in patients with a palliative outcome (*p* = 0.052). The latter result closely approaches statistical significance, suggesting that seizure disorder serves as an unfavorable prognostic marker; nevertheless, expanding the sample size is necessary to confirm these trends. In the studied cohort of patients (*n* = 21) with disseminated tuberculosis and CNS involvement, neurological complications predominated, with motor deficits (paresis/tetraparesis) and hydrocephalus being the most prevalent. Additionally, a substantial proportion of patients developed epileptic syndrome or sensory organ impairment (ophthalmic pathology and risk of hearing loss), emphasizing the severely debilitating nature of the disease course.

## 4. Discussion

The present study confirms that TBM remains a severe, debilitating disease characterized by an exceptionally high incidence of neurological complications. Of particular note is the low BCG vaccination coverage within the study cohort (only 48%), which represents a well-established risk factor for the development of TBM [9,10]. Although no statistically significant association was found between vaccination status and clinical outcomes (*p* = 0.266), a visible trend toward a more severe disease course was observed in unvaccinated individuals, particularly regarding seizure activity (*p* = 0.092). This underscores the crucial role of vaccination as a protective factor. The absence of reliable data regarding contact with tuberculosis patients in a substantial proportion of individuals highlights the significant contribution of hidden, undetected infectious sources, which complicates the timely identification of high-risk groups and implementation of preventive measures.

In this study, the initial clinical course of tuberculous meningitis was frequently characterized by attenuated classic meningeal symptoms, with meningeal signs documented at admission in only 38% of patients. In infants and young children, diagnostic confirmation was further confounded by the predominance of unique meningeal syndrome equivalents, manifesting as seizures or paroxysmal events.

The difficulty of timely TBM diagnosis was also driven by immune anergy, which may reduce the sensitivity of immunodiagnostic tests. The Mantoux test (2 TU) yielded negative results in nearly one-quarter of patients (22.3%), while the RTA skin test was negative in 40%. These findings highlight the persistent challenge of false-negative results in microbiological TB diagnostics, even with advanced methods such as PCR and the BACTEC system [11,12,13]. Under these circumstances, a comprehensive assessment integrating neuroimaging findings, clinical presentation, and epidemiological history becomes imperative.

Furthermore, the high prevalence of DR represents a highly alarming factor. The cumulative proportion of patients with MDR, pre-XDR, and XDR strains accounted for 47% of those with a definitively identified pathogen. The presence of XDR-TB, including resistance to bedaquiline and linezolid, represents a major therapeutic challenge, as it markedly limits treatment options and may worsen outcomes due to the poor BBB penetration of many second-line and reserve drugs [8,15,16].

Outcome analysis revealed a frequency of disabling complications. Motor deficits, hydrocephalus (with several cases requiring neurosurgical intervention), and epileptic syndrome emerged as the primary sequelae of the experienced meningoencephalitis. Statistical analysis demonstrated a trend toward an association between seizure disorder as a primary marker of encephalitis, as its presence could be closely associated with critically severe outcomes (palliative care or persistent vegetative state) (*p* = 0.052). However, larger studies are required to conclusively establish this relationship. Although the confidence interval for the odds ratio (*OR* = 24) was wide due to the limited sample size, this trend fundamentally aligns with clinical logic, according to which refractory seizures exacerbate hypoxia and secondary brain injury.

Several important methodological limitations should be considered when interpreting these findings. First, the retrospective design and small sample size (*n* = 21) inherently limit the statistical power of comparative analyses, resulting in wide confidence intervals (such as for seizure odds ratios) and precluding multivariable regression modeling. Second, there is a pronounced tertiary referral bias; our institution functions as a national reference and telemedicine hub, which enriches the cohort with unusually severe, complicated, or drug-resistant cases that may not represent the broader pediatric TBM population in community settings. Third, long-term post-discharge functional, neurodevelopmental, and rehabilitation outcomes beyond the acute treatment phase could not be systematically captured for patients evaluated via regional telemedicine. Fourth, pre-admission immunodiagnostic testing was performed inconsistently across referring centers. Future prospective, multicenter pediatric registries are necessary to validate these observations.

## 5. Conclusions

- Infants and young children who are unvaccinated with BCG represent a vulnerable group at risk for severe disseminated forms of tuberculosis, including meningitis. Routine immunodiagnostic screening should be strictly maintained in this population.
- The emergence of meningeal irritation or unexplained encephalopathy in BCG-unvaccinated children warrants urgent, comprehensive screening for tuberculosis.
- In this tertiary pediatric cohort, tuberculous meningitis presented with a critically severe meningoencephalitic course characterized by high rates of multiorgan dissemination (95%) and disabling complications, predominantly hydrocephalus (47.6%) and motor deficits (38.1%). In 19.0% of cases, irreversible brain damage necessitated transition to palliative care.
- Seizure activity during the acute phase demonstrated an exploratory association with unfavorable outcomes (*p* = 0.052), highlighting the clinical importance of continuous neurophysiological monitoring and aggressive seizure control.
- Diagnostic delays exceeding 14 days were descriptively associated with poorer clinical recovery. Given the high prevalence of drug-resistant strains (47.4%) and atypical blunted presentations at onset, early molecular CSF diagnostics and heightened clinical suspicion in primary pediatric care remain paramount.

## Figures and Tables

**Table 1:** Clinical and demographic characteristics of the patients (*n* = 21).

Characteristic	0–3 Years (*n* = 11)	4–6 Years (*n* = 3)	7–11 Years (*n* = 2)	12–17 Years (*n* = 5)	Total (*n* = 21)
Gender (M/F)	5 (45%)/6 (55%)	2 (67%)/1 (33%)	1 (50%)/1 (50%)	1 (20%)/4 (80%)	9 (43%)/12 (57%)
BCG vaccination status					
Vaccinated	2 (18.2%)	3 (100%)	2 (100%)	3 (60.0%)	10 (47.6%)
Unvaccinated	9 (81.8%)	0 (0%)	0 (0%)	2 (40.0%)	11 (52.4%)
History of TB contact					
Confirmed contact	10 (90.9%)	1 (33.3%)	1 (50.0%)	1 (20.0%)	13 (61.9%)

**Table 2:** Analysis of patient complaints at initial medical presentation (*n* = 21).

Clinical Manifestation	Number of Patients (*n* = 21)	(%)
Fever (elevated body temperature)	14	67
Fatigue, lethargy, malaise	9	43
Nausea and/or vomiting	11	52
Cephalgia	7	33
Cough, rhinorrhea, and upper respiratory tract symptoms	5	24
Altered level of consciousness (drowsiness, lethargy, loss of consciousness)	6	29
Abdominal pain and dyspepsia	11	52
Loss of appetite and food refusal	4	19
Seizures (including focal seizures)	4	19
Motor impairments (weakness in limbs, gait instability)	8	38
Meningeal signs (nuchal rigidity, hyperesthesia)	8	38
Other (pallor, cyanosis, hypernasality, weight loss, etc.)	5	24

**Table 3:** Drug resistance spectrum of *Mycobacterium tuberculosis* in the study cohort.

Resistance Pattern	Clinical Profile and Resistance Definition	Confirmed Cases (*n* = 19), *n* (%)	Total Cohort (*n* = 21), *n* (%)
DS	Drug-susceptible to all first-line agents	7 (36.8%)	7 (33.3%)
MDR	Resistance to at least Isoniazid (H) and Rifampicin (R)	3 (15.8%)	3 (14.3%)
Pre-XDR	MDR + resistance to any fluoroquinolone (FQ)	3 (15.8%)	3 (14.3%)
XDR	MDR + FQ resistance + resistance to at least one Group A drug (Bedaquiline/Linezolid)	3 (15.8%)	3 (14.3%)
Pending/ND	Culture in progress or DST unavailable	3 (15.8%)	3 (14.3%)
Total Confirmed	Definite microbiological identification	19 (100.0%)	19 (90.5%)

**Table 4:** Time to diagnosis verification, BCG vaccination status, and treatment outcomes.

Time to Final Diagnosis (Mean, Days)	Number of Patients, *n* (%)	BCG Vaccination, *n* (%)	Outcomes at the End of Treatment, *n* (%)
Up to 7 days	5 (24%)	2 (40%)	Clinical improvement—4 (80.0%); Persistent neurological impairment—1 (20.0%)
7 to 14 days	4 (19%)	1 (25%)	Clinical improvement—3 (75.0%); Persistent neurological impairment—1 (25.0%)
Over 14 days	12 (57%)	7 (58%)	Transfer to palliative care (critical irreversible brain damage)—4 (33.3%); Persistent neurological impairment—4 (33.3%); Clinical improvement—4 (33.3%)

## Data Availability

The original contributions presented in this study are included in the article. Further inquiries can be directed to the corresponding author.
